# Laparoscopic removal of retrievable inferior vena cava filters after failed or anticipated technically challenging endovascular retrieval: a retrospective case series of 28 patients

**DOI:** 10.3389/fphys.2026.1789114

**Published:** 2026-06-02

**Authors:** Peng Jiang, Jianlong Liu, Wei Jia, Pengji Gao, Yunxin Zhang, Xuan Tian, Zhiyuan Cheng, Jinyong Li, Xiao Liu, Mi Zhou, Chengjia Qu, Run Hua, Zhicun Li, Chenyang Tian

**Affiliations:** 1 Department of Vascular Surgery, Beijing Jishuitan Hospital, Capital Medical University, Beijing, China; 2 Department of General Surgery, Beijing Jishuitan Hospital, Capital Medical University, Beijing, China

**Keywords:** a retrospective case series, deep vein thromboembolism, failed or anticipated technically challenging endovascular retrieval, inferior vena cava filter, laparoscopy

## Abstract

**Background:**

Laparoscopic retrieval of inferior vena cava filters (IVCF) offers a minimally invasive alternative for patients with failed percutaneous retrieval or anticipated technically challenging endovascular retrieval, but systematic evaluations are limited.

**Methods:**

We retrospectively analyzed 28 patients who underwent surgical retrieval of IVCFs after failed endovascular retrieval or after preoperative imaging suggested anticipated technically challenging or potentially unsafe endovascular retrieval. Surgical techniques, perioperative outcomes, and a sensitivity analysis were described.

**Results:**

Laparoscopic retrieval was attempted in all 28 patients and completed laparoscopically in 25 patients, yielding a laparoscopic completion rate of 89.3%. Three patients required conversion to open surgery, corresponding to a conversion rate of 10.7%. Complete filter removal was achieved in 27 patients, resulting in an overall technical success rate of 96.4% when open conversion cases were included. One patient experienced intraoperative IVCF injury with massive hemorrhage and died in the early postoperative period despite conversion to open surgery, vascular repair, transfusion, and intensive care support. No routine caval occlusion was required in completed laparoscopic cases. During follow-up, no recurrent pulmonary embolism, symptomatic IVCF occlusion, or procedure-related late mortality was observed among surviving patients.

**Conclusions:**

Laparoscopic IVCF retrieval may be a feasible second-line option in selected patients with failed or anticipated technically challenging endovascular retrieval. The laparoscopic completion rate should be distinguished from the overall technical success rate, which includes cases completed after open conversion. Because this study lacked a comparator group, these findings should be interpreted as descriptive and should not be used to infer superiority over open surgery or advanced endovascular techniques.

## 1 Introduction

The Inferior Vena Cava Filter (IVCF) constitutes a critical medical device for the prevention of fatal Pulmonary Embolism (PE), particularly in patients with Venous Thromboembolism (VTE) who present with contraindications to anticoagulation or for whom anticoagulant therapy has proven ineffective (Duffett and Carrier, 2017). However, the permanent retention of these filters is associated with a spectrum of long-term complications, including filter migration, fracture, perforation of the inferior vena cava, thrombus formation, and occlusion (Ramakrishnan et al., 2023). Consequently, the 2020 Society of Interventional Radiology (SIR) Clinical Practice Guideline recommends structured follow-up after IVCF placement and retrieval or conversion of retrievable filters once the risk of PE has resolved, unless the risk of removal outweighs the expected benefit (Kaufman et al., 2020).

Despite the widespread application of IVCF, it remains technically challenging in a substantial proportion of patients. A systematic review and meta-analysis reported a pooled success rate of 76% (95% CI, 65%–84%) for standard retrieval techniques and 90% (95% CI, 82%–94%) for advanced retrieval techniques, supporting the need for individualized escalation strategies when standard snare retrieval is unlikely to succeed or has failed (Merritt et al., 2022). The likelihood of retrieval difficulty increases with prolonged dwell time and unfavorable anatomical features, including filter tilt, hook embedding, strut penetration through the caval wall, and device deformation (Jia et al., 2015; Kang et al., 2023; Kethidi et al., 2023). A 2023 systematic review of advanced IVCF retrieval reported that complex retrieval was most commonly performed after failed standard retrieval or in the presence of filter tilt or caval wall embedding, with a pooled advanced retrieval success rate of 92.6% and a major complication rate of 2.8% (Kethidi et al., 2023). In addition, a multicenter study found that hook embedment and strut penetration were predictors of snare retrieval failure, supporting the role of preprocedural imaging in selecting retrieval strategies (Kang et al., 2023). When endovascular retrieval attempts fail, long-term filter retention exposes patients to serious risks, including caval perforation, strut fracture, filter migration, and potential injury to adjacent critical structures such as the small bowel, duodenum, or aorta. Therefore, timely and safe retrieval of these “trapped” filters is paramount to avert subsequent life-threatening sequelae.

Although standard and advanced endovascular techniques, including loop-snare, forceps-assisted, and laser-assisted retrieval, have expanded the treatment options for complex IVCF retrieval (Kuyumcu and Walker, 2016; Kethidi et al., 2023; Swersky and Desai, 2024), selected patients with extraluminal penetration, adjacent-organ involvement, or anatomy considered unsuitable for safe endovascular retrieval may still require surgical management. In such cases, laparoscopic retrieval may provide direct visualization for adhesiolysis, management of extraluminal filter components, and controlled venotomy for filter removal (Benrashid et al., 2015; Wang H. et al., 2021; Cheng et al., 2023). Open surgical retrieval has been reported as a salvage option after failed endovascular retrieval, but available data remain limited and largely consist of retrospective series (Tian et al., 2023).

While laparoscopic retrieval is increasingly recognized as a less invasive surgical option for selected patients with failed endovascular retrieval or anticipated technically challenging endovascular retrieval, there remains a paucity of systematic case series comprehensively evaluating this approach. This study analyzes 28 cases from our institution in which laparoscopic retrieval was employed after failed endovascular retrieval or direct surgical referral based on preoperative assessment of technically challenging or potentially unsafe endovascular retrieval. Our objective was to describe the technical success rate, laparoscopic completion rate, perioperative outcomes, and short-term follow-up findings of this strategy in selected patients with failed or anticipated difficult retrieval of IVC filters.

## 2 Methods

### 2.1 Baseline characteristics

Between April 2023 and November 2025, our institution managed 28 consecutive patients who underwent surgical removal of an IVCF because of either failed percutaneous/endovascular retrieval or anticipated technically challenging endovascular retrieval based on preoperative imaging and multidisciplinary assessment by vascular surgeons and interventional specialists. The median age was 62 years (range, 30–79 years), and the cohort comprised 13 men and 15 women. All patients had been implanted with a retrievable IVCF. The indwelling time was at least 1 month, with a mean duration of 4.6 months. Preoperative contrast-enhanced CT venography (CTV) showed strut and/or hook penetration through the IVC wall in most patients. Notably, in the three patients who required conversion to open surgery, the retrieval hook did not completely penetrate the caval wall, which precluded adequate laparoscopic exposure and safe control of the filter apex; in some of these cases, the hook was also oriented posteriorly, further increasing the technical difficulty. Patients were eligible for inclusion if they underwent laparoscopic or open surgical removal of a retrievable IVC filter because of either failed standard percutaneous/endovascular retrieval or anticipated technically challenging endovascular retrieval. Among the 28 patients, 23 had undergone prior failed endovascular retrieval attempts, whereas 5 had no prior retrieval attempt but were referred directly for surgical retrieval because preoperative imaging suggested anticipated technically challenging or potentially high-risk endovascular retrieval. The number of prior failed retrieval attempts was zero in 5 patients, one in 12 patients, two in 9 patients, and three in 2 patients.

For completeness, we recorded the filter model/type (Octoparms, n = 10; Denali, n = 7; Option, n = 7; Celect, n = 4), thrombus location (data not available), and major predisposing factors for lower-extremity DVT [fracture/orthopedic trauma, n = 9; malignancy, n = 2; immobilization, n = 1; post-surgery (non-orthopedic), n = 3; other/unknown, n = 13]. These characteristics are summarized in **Table 1**. Anticipated technically challenging endovascular retrieval was defined as the presence of one or more unfavorable preoperative imaging findings, including severe filter tilt, embedded or unfavorably oriented retrieval hook, strut or hook penetration beyond the caval wall, close proximity to or involvement of adjacent organs, suspected filter fracture, or other anatomical features that made standard endovascular retrieval unlikely to succeed or potentially unsafe. For patients without prior retrieval attempts, the decision to proceed directly to laparoscopic retrieval was made after multidisciplinary review of the imaging findings and procedural risks.

**Table 1:** Baseline characteristics of the 28 patients.

Variable	Value
No. of patients	28
Age, median (range), years	62 (30–79)
Sex	
Male	13 (46.4%)
Female	15 (53.6%)
Filter indwelling time, mean, months	4.6
Filter model/type	
Octoparms	10 (35.7%)
Denali	7 (25.0%)
Option	7 (25.0%)
Celect	4 (14.3%)
Preoperative retrieval pathway	
Failed prior endovascular retrieval	23 (82.1%)
Direct surgical referral due to anticipated technically challenging endovascular retrieval	5 (17.9%)
Preoperative imaging findings	
Strut and/or hook penetration beyond the caval wall	12 (70.6%)
Severe filter tilt or embedded/unfavorably oriented retrieval hook	8 (47.1%)
Close proximity to or suspected involvement of adjacent organs	4 (23.5%)
High filter position or posteriorly oriented hook limiting safe endovascular access	3 (17.6%)
Suspected filter fracture or structural deformation	1 (5.9%)
No. of prior failed endovascular retrieval attempts	
0 attempts	5 (17.9%)
1 attempt	12 (42.9%)
2 attempts	9 (32.1%)
3 attempts	2 (7.1%)
Major predisposing factors for DVT	
Fracture/orthopedic trauma	9 (32.1%)
Malignancy	2 (7.1%)
Immobilization	1 (3.6%)
Post-surgery, non-orthopedic	3 (10.7%)
Other/unknown	13 (46.4%)
Preoperative thrombus location	Not available in 28/28 (100%)

Data are presented as n (%) unless otherwise indicated. Patients were divided into two preoperative retrieval pathways: failed prior endovascular retrieval and direct surgical referral due to anticipated technically challenging endovascular retrieval. The latter referred to patients without a prior retrieval attempt who were selected for surgical retrieval because preoperative imaging suggested unfavorable anatomical features that made standard endovascular retrieval unlikely to succeed or potentially unsafe. Reasons for direct surgical referral were not mutually exclusive, and some patients had more than one unfavorable imaging feature. Preoperative thrombus location was not available in the source records for all 28 patients and was therefore reported as missing without imputation. DVT, deep vein thrombosis.

### 2.2 Surgical technique

All procedures were performed under general anesthesia with all patients in the supine position (no lateral decubitus positioning). A transperitoneal laparoscopic approach was used in all cases. After establishing pneumoperitoneum, 4–5 trocar ports were placed. The abdominal cavity was explored, and adhesiolysis was performed when necessary.

The infrarenal IVC was approached mainly by Kocher mobilization of the duodenum. When operating adjacent to the duodenum and pancreatic head, dissection was carried out layer-by-layer along the avascular fusion fascia plane to expose the IVC and the filter penetration area. Surrounding structures (duodenum, pancreas, kidney, and ureter) were carefully protected. In the presence of fibrosis or granulation tissue, dissection was performed predominantly bluntly with selective sharp dissection to avoid traction-related caval wall tears. IVC clamping/occlusion was not routinely performed in our laparoscopic cases, and complete circumferential isolation of the IVC was not required.

The key operative strategy was targeted exposure of the retrieval hook and its penetration point. After adequate exposure, a 5–0 nonabsorbable purse-string, U-shaped, or figure-of-eight suture was placed around the penetration site and left untied. A retrieval set was then introduced through a trocar; under direct laparoscopic visualization, the hook was snared, the filter was sequentially collapsed into the sheath, and the filter was removed en bloc through the limited venotomy or penetration site. The suture was then tightened to close the caval wall defect, and the operative field was irrigated and inspected. Minor bleeding was controlled with laparoscopic compression and additional reinforcement sutures when necessary.

**Figure 1:**
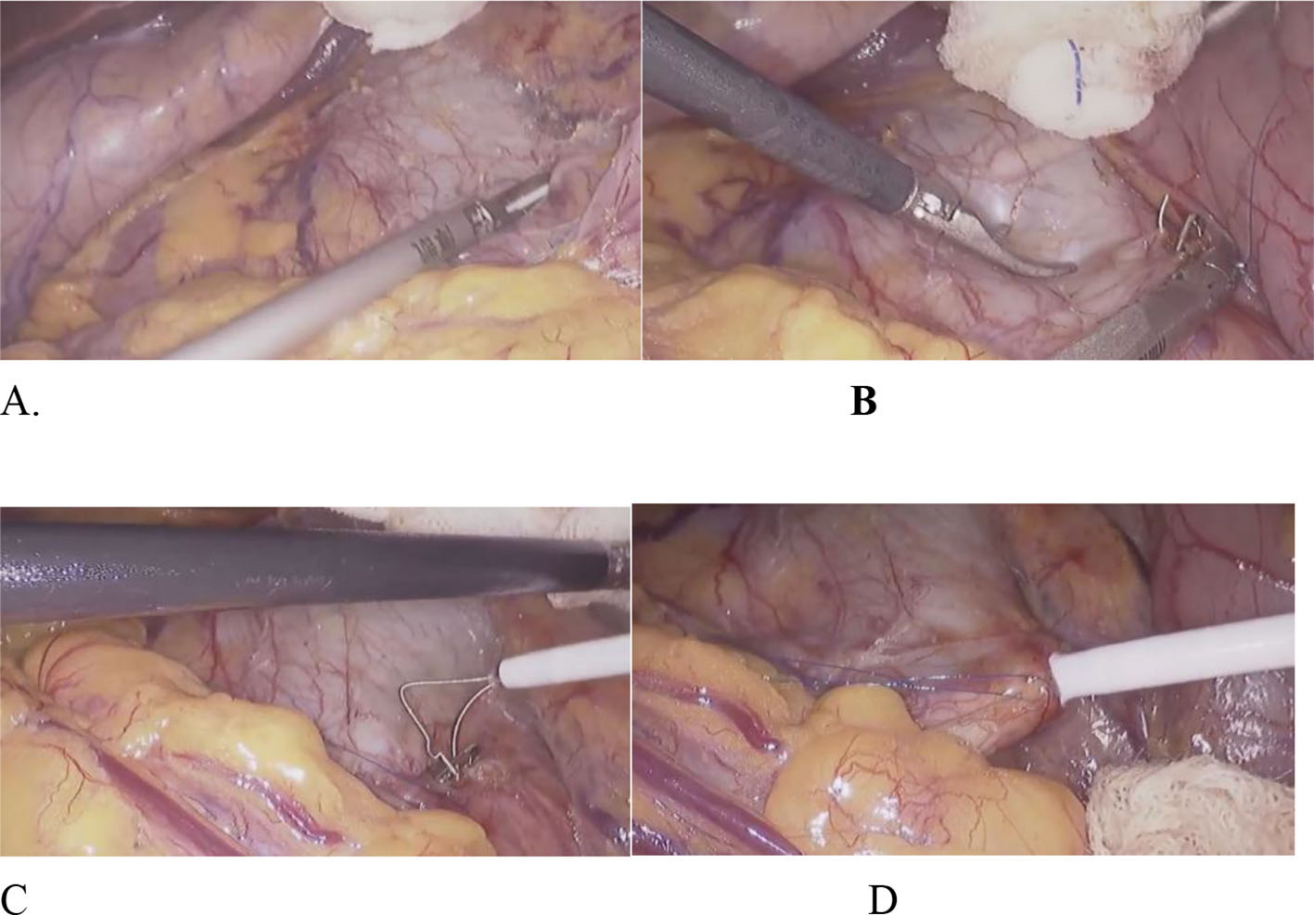
Key surgical steps of laparoscopic endocaval retrieval of an inferior vena cava filter. **(A)** Careful dissection to expose the local segment of the inferior vena cava. **(B)** Exposure of the retrieval hook, with placement of a U-shaped or purse-string suture around the penetration site before extraction. **(C)** Snaring of the IVCF hook using a retrieval set under direct laparoscopic visualization. **(D)** After IVCF through the sheath, the suture is tightened and knotted to close the caval wall defect. IVC, inferior vena cava.

Conversion to open surgery was undertaken when laparoscopic exposure of the hook was unsafe or inadequate (e.g., the hook did not completely penetrate the IVC wall and/or was oriented posteriorly) or when bleeding could not be controlled laparoscopically. Open removal and venous repair were performed via a right rectus abdominis incision or a right subcostal oblique incision, following the same principles of targeted exposure, extraction, and immediate defect closure. The key surgical steps of laparoscopic endocaval retrieval of an inferior vena cava filter are shown in **Figure 1**.

### 2.3 Postoperative management and follow-up

Postoperatively, all patients received close monitoring of vital signs, abdominal examination findings, drainage output, hemoglobin levels, coagulation parameters, and distal lower limb perfusion either in the intensive care unit or in a specialized vascular surgical ward. Patients with hemodynamic instability, major bleeding, open conversion, or other high-risk perioperative features were admitted to the intensive care unit. Therapeutic anticoagulation was routinely re-initiated within 24 to 48 hours after surgery, provided that hemodynamic stability had been achieved and no contraindication to anticoagulation was present. Postoperative complications were recorded during hospitalization and follow-up and were graded according to the Clavien-Dindo classification. Major complications were defined as Clavien-Dindo grade III or higher.

Follow-up was performed through outpatient visits, telephone interviews, and imaging review. Duplex ultrasonography or CT venography was used to assess IVC patency, recurrent thrombosis, retroperitoneal hematoma, pseudoaneurysm, and other procedure-related complications. Follow-up outcomes included survival status, recurrent deep vein thrombosis or pulmonary embolism, symptomatic IVC stenosis or occlusion, reintervention, and late procedure-related complications.

### 2.4 Statistical analysis

All analyses were descriptive because of the retrospective case-series design and limited sample size. Continuous variables were reported as median and range, and categorical variables were reported as counts, percentages, and 95% confidence intervals where appropriate. For key binary outcomes, including overall technical success, laparoscopic completion, conversion to open surgery, procedure-related mortality, major complications, IVC patency, recurrent deep vein thrombosis, and recurrent pulmonary embolism, 95% confidence intervals were calculated using the Wilson method.

Overall technical success was defined as complete filter removal, whether achieved laparoscopically or after conversion to open surgery. Laparoscopic completion was defined as complete filter removal without conversion to open surgery. Conversion rate was defined as the proportion of patients in whom the initial laparoscopic procedure was converted to open surgery. Procedure-related mortality was defined as death occurring intraoperatively or during the postoperative hospitalization that was judged to be directly or probably related to the retrieval procedure or its complications.

A sensitivity analysis was conducted by excluding the single procedure-related mortality case to evaluate whether the main technical and perioperative estimates were disproportionately influenced by this outlier event. Outcomes assessed in the sensitivity analysis included operative time, estimated blood loss, overall technical success, laparoscopic completion, conversion to open surgery, and mortality. In addition, a leave-one-out analysis was performed for operative time and estimated blood loss to assess the stability of the median values after excluding each patient one at a time.

Given the small sample size, no formal hypothesis testing or multivariable modeling was performed. Potential factors associated with prolonged operative time, including filter indwelling time, filter type, caval wall penetration, retrieval hook orientation, and conversion to open surgery, were explored descriptively only. Missing data were reported explicitly for each variable when present. No imputation was performed, and variables with completely unavailable data, such as preoperative thrombus location, were not included in descriptive subgroup summaries or exploratory assessments. All statistical analyses were performed using Microsoft Excel (Microsoft Corp., Redmond, WA, USA) and Python (version 3.11.6; Python Software Foundation).

### 2.5 Ethics statement

This retrospective study was approved by the Institutional Review Board of Beijing Jishuitan Hospital, Capital Medical University (Approval No. K-2024-161-00). The requirement for written informed consent for study participation was waived by the Institutional Review Board because of the retrospective nature of the study and the use of de-identified clinical data. All patients provided written informed consent for the surgical procedure as part of routine clinical care. The study was conducted in accordance with the principles of the Declaration of Helsinki.

## 3 Results

### 3.1 Surgical outcomes

Of the 28 patients, 23 undergone prior failed endovascular retrieval attempts, whereas 5 had not undergone a prior retrieval attempt but were referred directly for surgical retrieval because of anticipated technically challenging or potentially unsafe endovascular retrieval based on preoperative imaging. Laparoscopic retrieval was attempted in all 28 patients. Complete filter removal was achieved laparoscopically in 25 patients, yielding a laparoscopic completion rate of 89.3% (25/28; 95% CI, 72.8%–96.3%). Three patients required conversion to open surgery, corresponding to a conversion rate of 10.7% (3/28; 95% CI, 3.7%–27.2%). Among these three conversion cases, complete filter removal was achieved after conversion in two patients, whereas retrieval was aborted in one patient because of uncontrolled hemorrhage after intraoperative IVC injury. Therefore, the overall technical success rate, including cases completed after open conversion, was 96.4% (27/28; 95% CI, 82.3%–99.4%). The median operative time was 120 minutes (range, 38–222 minutes), and the median estimated blood loss was 50 mL (range, 10–1500 mL).

One laparoscopic procedure was terminated because of substantial hemorrhage after intraoperative IVC injury. This patient was the only mortality case in the cohort, corresponding to a procedure-related mortality rate of 3.6% (1/28; 95% CI, 0.6%–17.7%). The filter was positioned relatively high, and the retrieval hook was oriented posteriorly, which resulted in inadequate laparoscopic exposure and difficult control of the filter apex. After IVC injury and massive hemorrhage occurred, the procedure was converted to open surgery for hemostasis and vascular repair. The estimated blood loss was 1500 mL, and IVCF was aborted. The patient received blood transfusion, vasoactive support, and postoperative intensive care treatment. Despite these measures, the patient died in the early postoperative period. The cause of death was considered procedure-related hemorrhagic shock with subsequent multiorgan dysfunction.

### 3.2 Postoperative outcomes and follow-up

Postoperative outcomes are summarized in **Supplementary Table 1**. Among the 27 patients in whom IVCF was completed, no patient required reoperation for postoperative bleeding, pseudoaneurysm, bowel injury, or ureteral injury. One patient experienced procedure-related death after intraoperative IVC injury and massive hemorrhage, which was classified as a Clavien-Dindo grade V complication. The remaining postoperative complications were mild and were managed conservatively. No surviving patient developed symptomatic pulmonary embolism, clinically evident recurrent lower-extremity deep vein thrombosis, or symptomatic IVC occlusion during follow-up.

ICU admission was required in 4 patients, including the mortality case and patients who underwent open conversion or required close hemodynamic monitoring. Blood transfusion was administered in 3 patients, including the patient with massive hemorrhage. The median postoperative hospital stay was 7 days (range, 4–18 days). Follow-up was available for 27 patients, with a median follow-up duration of 8 months (range, 1–24 months). Follow-up imaging with duplex ultrasonography or CT venography demonstrated preserved IVC patency in 26 of 27 surviving patients. One patient had asymptomatic mild IVC stenosis without the need for reintervention. No late filter-related complications or procedure-related late deaths were observed.

### 3.3 Sensitivity analysis

**Figure 2:**
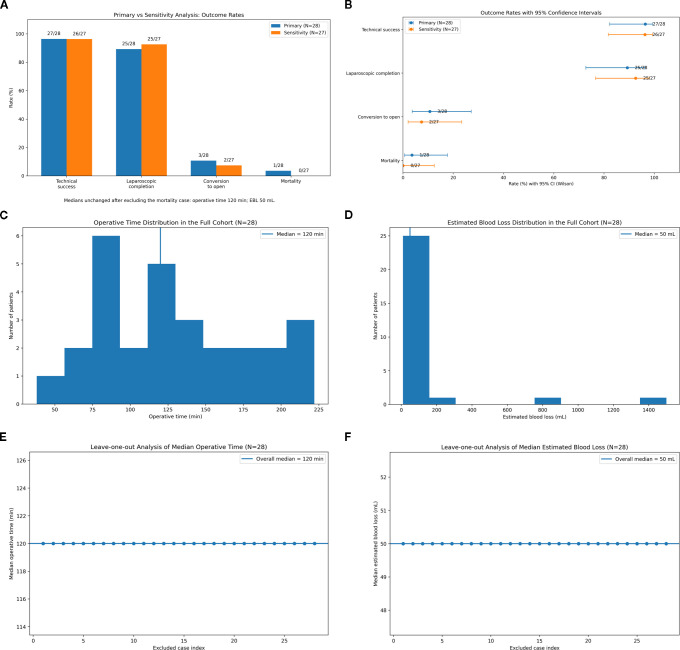
Sensitivity analysis excluding the single procedure-related mortality case and leave-one-out assessment of perioperative outcomes. **(A)** Comparison of key outcome rates between the primary cohort (N = 28) and the sensitivity cohort excluding the mortality case (N = 27), including overall technical success, laparoscopic completion, conversion to open surgery, and procedure-related mortality. **(B)** Outcome rates with 95% confidence intervals calculated using the Wilson method. **(C)** Distribution of operative time in the full cohort (N = 28), with the median indicated. **(D)** Distribution of estimated blood loss in the full cohort (N = 28), with the median indicated. **(E)** Leave-one-out analysis showing the stability of the median operative time after excluding each patient one at a time. **(F)** Leave-one-out analysis showing the stability of the median estimated blood loss after excluding each patient one at a time. EBL, estimated blood loss; CI, confidence interval.

To assess the influence of the single procedure-related mortality case on the perioperative estimates, we performed a sensitivity analysis excluding this patient. After exclusion, the median operative time remained 120 minutes (range, 38–222 minutes), and the median estimated blood loss remained 50 mL, whereas the range decreased from 10–1500 mL in the full cohort to 10–800 mL after exclusion of the mortality case. The overall technical success rate was 96.3% (26/27; 95% CI, 81.7%–99.3%), compared with 96.4% (27/28; 95% CI, 82.3%–99.4%) in the full cohort. The laparoscopic completion rate was 92.6% (25/27; 95% CI, 76.6%–97.9%), compared with 89.3% (25/28; 95% CI, 72.8%–96.3%) in the full cohort. The conversion-to-open rate was 7.4% (2/27; 95% CI, 2.1%–23.4%), compared with 10.7% (3/28; 95% CI, 3.7%–27.2%) in the full cohort. Procedure-related mortality decreased from 3.6% (1/28; 95% CI, 0.6%–17.7%) to 0% (0/27; 95% CI, 0%–12.5%). These findings suggest that the main technical outcomes were not materially altered by exclusion of the mortality case; however, the mortality case remains clinically important for safety interpretation and should not be minimized by the sensitivity analysis (**Table 2**). The sensitivity analysis and leave-one-out assessment of median operative time and estimated blood loss are shown in **Figure 2**.

**Table 2:** Sensitivity analysis excluding the single procedure-related mortality case.

Outcome	Primary analysis (N = 28)	Sensitivity analysis (N = 27)*
Operative time, median (range), min	120 (38–222)	120 (38–222)
Estimated blood loss, median (range), mL	50 (10–1500)	50 (10–800)
Technical success of retrieval, n/N (%)	27/28 (96.4)	26/27 (96.3)
Laparoscopic completion, n/N (%)	25/28 (89.3)	25/27 (92.6)
Conversion to open surgery, n/N (%)	3/28 (10.7)	2/27 (7.4)
Procedure-related mortality, n/N (%)	1/28 (3.6)	0/27 (0)

*Sensitivity analysis excludes the single procedure-related mortality case with intraoperative IVC injury and massive hemorrhage. IVC, inferior vena cava.

## 4 Discussion

This study presents a cohort of 28 patients who underwent laparoscopic or open surgical retrieval of IVC filters because of failed percutaneous/endovascular retrieval or anticipated technically challenging endovascular retrieval. Laparoscopic retrieval was completed in 25 patients, corresponding to a laparoscopic completion rate of 89.3%, whereas 3 patients required conversion to open surgery. The overall technical success rate was 96.4% when open conversion cases were included. These results suggest that laparoscopic surgery may be considered as a minimally invasive option in selected complex cases characterized by filter penetration through the caval wall, significant perifilter adhesions, embedded or unfavorably oriented retrieval hooks, or anticipated difficulty with standard endovascular retrieval. A notable technical feature in our practice is the pre-placement of a purse-string/figure-of-eight suture around the hook penetration site before extraction. This allows immediate closure of the caval wall defect once the filter is removed and, together with laparoscopic compression hemostasis, may help reduce operative bleeding and shorten operative time, especially in cases with dense adhesions or limited working space. Importantly, the overall technical success rate should not be interpreted as the success rate of laparoscopy alone. Rather, it reflects the combined success of an initial laparoscopic strategy with open conversion when necessary. This distinction is clinically relevant because conversion to open surgery represents an important safety measure rather than a technical failure in all cases. Therefore, both the laparoscopic completion rate and the overall technical success rate should be reported when evaluating this approach.

The single mortality case deserves specific consideration. This patient had a relatively high filter position and a posteriorly oriented retrieval hook, which limited laparoscopic exposure and safe control of the filter apex. Intraoperative IVC injury resulted in massive hemorrhage, requiring conversion to open surgery, vascular repair, transfusion, and intensive care treatment. The patient died in the early postoperative period, and the death was considered procedure-related. This case highlights that laparoscopic retrieval, although feasible in selected patients, carries potentially serious vascular risks. Unfavorable hook orientation, high filter position, dense adhesions, and inadequate exposure should prompt early consideration of open conversion or planned open retrieval.

In terms of postoperative outcomes, most surviving patients recovered without major complications. Follow-up imaging showed preserved IVC patency in the majority of surviving patients, and no recurrent pulmonary embolism, symptomatic IVC occlusion, or late procedure-related death was observed during follow-up. Nevertheless, the relatively short and variable follow-up duration limits the ability to draw definitive conclusions regarding long-term venous patency, recurrent thrombosis, or delayed complications. Future studies should incorporate standardized imaging follow-up and longer observation periods.

Prolonged IVC filter dwell time and unfavorable imaging features are associated with increasing retrieval complexity. Prior reviews have shown that hook embedding, strut penetration, filter tilt, and device deformation are common reasons for standard retrieval failure and often prompt escalation to advanced endovascular techniques (Merritt et al., 2022; Kang et al., 2023; Kethidi et al., 2023; Swersky and Desai, 2024). In the 2023 systematic review by Kethidi et al., advanced retrieval techniques were used primarily after failed standard retrieval or in cases with tilt or caval wall embedding, achieving a pooled success rate of 92.6% with a major complication rate of 2.8% (Kethidi et al., 2023). Pratt et al. (2020) reported that asymptomatic patients with unsuccessful percutaneous IVCF retrieval rarely developed complications during midterm follow-up despite significant strut penetration through the caval wall. They also suggested that, in the absence of symptoms or other complications, open surgical removal may not be necessary, whereas symptomatic or complicated cases may benefit from percutaneous or open filter removal. In our series, the mean filter indwelling time was 4.6 months. In most patients, preoperative imaging suggested caval wall penetration or other unfavorable anatomical features. Twenty-three patients had experienced prior failed endovascular retrieval attempts, whereas 5 were referred directly for surgical retrieval because standard endovascular retrieval was considered technically challenging or potentially unsafe. This decision pathway is consistent with recent endovascular retrieval literature emphasizing that preprocedural imaging findings, particularly hook embedment and strut penetration, can predict snare retrieval failure and may justify an individualized retrieval strategy rather than repeated standard snare attempts (Kang et al., 2023; Swersky and Desai, 2024).The laparoscopic approach provides distinct advantages in this setting: it allows for direct visualization to meticulously dissect adhesions, facilitates controlled isolation of the IVC for potential vascular occlusion, and enables the precise management of filter components that have penetrated into surrounding viscera, thereby minimizing the risk of iatrogenic vascular or visceral injury associated with blind endovascular maneuvers. Our findings should also be interpreted alongside the broader literature on advanced endovascular retrieval. Systematic reviews have reported high technical success rates for advanced techniques, including loop-snare, forceps-assisted, and laser-assisted retrieval, but these approaches remain operator-dependent and are generally concentrated in experienced centers (Merritt et al., 2022; Kethidi et al., 2023). Therefore, laparoscopic retrieval should be viewed as one component of an individualized management algorithm rather than a replacement for advanced endovascular retrieval. It should be emphasized that the present cohort was not limited exclusively to patients after failed endovascular retrieval. A substantial proportion of patients were referred directly for surgical retrieval on the basis of preoperative imaging findings suggesting anticipated technically challenging or high-risk endovascular retrieval. Therefore, the findings of this study should be interpreted as applying to selected patients with either failed or anticipated difficult endovascular retrieval, rather than exclusively to patients after unsuccessful percutaneous attempts.

Historically, open surgical retrieval has been used as a salvage option for filters that cannot be removed endovascularly or when endovascular retrieval is considered unsafe (Tian et al., 2023). In our study, the laparoscopic completion rate was 89.3%, and the overall technical success rate increased to 96.4% after including conversions to open surgery. These descriptive results should be interpreted in the context of both open surgical series and contemporary advanced endovascular retrieval data, rather than as evidence of superiority or equivalence (Merritt et al., 2022; Kethidi et al., 2023; Tian et al., 2023). The laparoscopic approach may offer potential advantages related to reduced tissue trauma and postoperative recovery in selected patients, but these potential benefits require confirmation in comparative studies. However, in patients in whom the retrieval hook does not completely penetrate the caval wall or is directed toward the posterior IVC wall, laparoscopic exposure of the filter apex can be particularly challenging; in such situations, open surgery remains a safe and reliable alternative. When laparoscopic exposure is limited by obscured anatomy or dense adhesions, timely conversion to open surgery provides a dependable contingency plan.

The intraoperative strategy for vascular control during laparoscopic retrieval remains debated. In previous laparoscopic series, most selected cases were completed without routine caval occlusion; one 10-patient series reported that nine procedures were completed without occlusion and one required temporary occlusion, whereas a 7-patient retroperitoneal series reported no routine caval occlusion (Wang et al., 2020; Wang HD. et al., 2021).Consistent with these reports, none of the laparoscopic cases in our series required IVC occlusion.

Our technique emphasizes a “targeted, non-occlusive” strategy: (i) no routine IVC clamping/occlusion; (ii) limited dissection confined to the filter apex/retrieval hook region rather than circumferential isolation of the IVC; and (iii) pre-placement of a fine nonabsorbable purse-string/U-/figure-of-eight suture around the hook penetration site before extraction, which is immediately tightened and tied after the filter is removed. Together with laparoscopic compression using gauze/pledgets, this workflow enables rapid hemostasis for most bleeding encountered intraoperatively. These technical details represent features of our approach and may help limit dissection and facilitate hemostasis, although their impact on operative time, blood loss, or recovery cannot be determined without comparative data.

Nevertheless, vascular control and timely conversion to laparotomy should remain readily available when bleeding cannot be controlled laparoscopically or when safe laparoscopic exposure is not achievable, particularly in cases with unfavorable filter tilt or hook orientation (Wang et al., 2018).

In selected patients, laparoscopic surgery may offer a less invasive alternative to traditional open laparotomy. The magnified laparoscopic view may facilitate identification of anatomical planes and bleeding points during dissection (Trilling et al., 2021). Previous case reports have described the feasibility of laparoscopic or robotic-assisted surgical retrieval in selected patients after failed endovascular attempts or when filter components extended beyond the caval wall (Benrashid et al., 2015; Cheng et al., 2023). However, the present study was not designed to evaluate cost-effectiveness, quality of life, length of hospital stay, or comparative recovery outcomes. These observations suggest potential recovery-related advantages of a minimally invasive approach; however, the present study was not designed to evaluate cost-effectiveness, quality of life, or comparative recovery outcomes.

### 4.1 Limitations

This study has several limitations. First, it was a retrospective single-center case series with a small sample size, which limits the generalizability of the findings. Second, the study population was highly selected and included patients referred for surgical retrieval after failed endovascular retrieval or because endovascular retrieval was anticipated to be technically challenging or potentially unsafe; therefore, selection bias cannot be excluded. Third, the study lacked a comparator group, such as patients treated with open surgery, advanced endovascular retrieval techniques, or conservative management, and thus no conclusions can be drawn regarding the superiority or comparative effectiveness of laparoscopic retrieval. Fourth, the procedures were performed by an experienced surgical team, and the outcomes may be operator-dependent and not directly reproducible in centers with different levels of laparoscopic vascular experience. Fifth, the follow-up duration was relatively short and variable, limiting assessment of long-term IVC patency, recurrent thrombosis, and delayed procedure-related complications. Sixth, some clinical and imaging data were incomplete, including preoperative thrombus location and detailed anatomical predictors of technical difficulty. Finally, because of the limited sample size, potential factors associated with prolonged operative time or conversion to open surgery could only be explored descriptively and were not suitable for formal statistical modeling.

## 5 Conclusion

Laparoscopic retrieval may be a feasible second-line option in selected patients with IVCF after failed endovascular retrieval or when endovascular retrieval is anticipated to be technically challenging or potentially unsafe. Given that real-world IVCF retrieval rates remain suboptimal despite professional guidelines, strategies such as laparoscopic retrieval may represent an important option for selected filters that cannot be removed safely or successfully by percutaneous/endovascular approaches (Wu et al., 2025). In this descriptive case series, the laparoscopic completion rate was 89.3%, whereas the overall technical success rate was 96.4% when open conversion cases were included. These two outcomes should be interpreted separately. Because this study lacked a comparator group, the findings should not be interpreted as showing superiority over open surgery or advanced endovascular techniques. Careful patient selection, readiness for vascular control and open conversion, and standardized long-term follow-up remain essential.

## Data Availability

The raw data supporting the conclusions of this article will be made available by the authors, without undue reservation.

## Supplementary material

Supplementary Table 1 Perioperative and follow-up outcomes.
