# Supplementary material for: Laparoscopic removal of retrievable inferior vena cava filters after failed or anticipated technically challenging endovascular retrieval: a retrospective case series of 28 patients
Source: Front Physiol. 2026 Jun 2;17:1789114. doi: 10.3389/fphys.2026.1789114 (PMC13269042; doi:10.3389/fphys.2026.1789114)
Supplement: Supplementary Table 1 — Perioperative and follow-up outcomes. [file Table1.docx]

Table S1. Perioperative and Follow-up Outcomes

| **Outcome** | **Value** |
| --- | --- |
| **Laparoscopic retrieval attempted** | 28/28 (100%) |
| **Laparoscopic completion** | 25/28 (89.3%) |
| **Conversion to open surgery** | 3/28 (10.7%) |
| **Overall technical success, including open conversion** | 27/28 (96.4%) |
| **Failed retrieval / aborted procedure** | 1/28 (3.6%) |
| **Operative time, median (range), min** | 120 (38–222) |
| **Estimated blood loss, median (range), mL** | 50 (10–1500) |
| **Routine IVC clamping in completed laparoscopic cases** | 0/25 (0%) |
| **Intraoperative IVC injury with massive hemorrhage** | 1/28 (3.6%) |
| **Blood transfusion** | 3/28 (10.7%) |
| **ICU admission** | 4/28 (14.3%) |
| **Procedure-related mortality** | 1/28 (3.6%) |
| **Clavien-Dindo grade I–II complications** | 2/28 (7.1%) |
| **Clavien-Dindo grade III–IV complications** | 0/28 (0%) |
| **Clavien-Dindo grade V complications** | 1/28 (3.6%) |
| **Postoperative hospital stay, median (range), days** | 7 (4–18) |
| **Follow-up available** | 27/28 (96.4%) |
| **Follow-up duration, median (range), months** | 8 (1–24) |
| **Patent IVC on follow-up imaging among survivors** | 26/27 (96.3%) |
| **Asymptomatic mild IVC stenosis** | 1/27 (3.7%) |
| **Symptomatic IVC occlusion** | 0/27 (0%) |
| **Recurrent lower-extremity DVT** | 0/27 (0%) |
| **Recurrent pulmonary embolism** | 0/27 (0%) |
| **Late procedure-related death** | 0/27 (0%) |
| **Reintervention during follow-up** | 0/27 (0%) |

Note: Data are presented as n/N (%) unless otherwise indicated. Overall technical success included patients in whom complete filter removal was achieved laparoscopically or after conversion to open surgery. Laparoscopic completion was defined as complete filter removal without open conversion. The single Clavien-Dindo grade V complication was the procedure-related mortality case after intraoperative IVC injury and massive hemorrhage. For key binary outcomes, 95% confidence intervals were calculated using the Wilson method and are reported in the text or displayed in Figure 2. IVC, inferior vena cava; ICU, intensive care unit; DVT, deep vein thrombosis.
